# Deep learning to automatically evaluate HER2 gene amplification status from fluorescence in situ hybridization images

**DOI:** 10.1038/s41598-023-36811-z

**Published:** 2023-06-16

**Authors:** Tian Xue, Heng Chang, Min Ren, Haochen Wang, Yu Yang, Boyang Wang, Lei Lv, Licheng Tang, Chicheng Fu, Qu Fang, Chuan He, Xiaoli Zhu, Xiaoyan Zhou, Qianming Bai

**Affiliations:** 1 Department of Pathology, Fudan University Shanghai Cancer Centre, 270 Dong’an Road, Shanghai, 200032 China; 2 Department of Oncology, Shanghai Medical College Fudan University, Shanghai, China; 3 Shanghai Aitrox Technology Corporation Limited, Shanghai, China

**Keywords:** Cancer, Molecular medicine

## Abstract

Human epidermal growth factor receptor 2 (HER2) gene amplification helps identify breast cancer patients who may respond to targeted anti-HER2 therapy. This study aims to develop an automated method for quantifying HER2 fluorescence in situ hybridization (FISH) signals and improve the working efficiency of pathologists. An Aitrox artificial intelligence (AI) model based on deep learning was constructed, and a comparison between the AI model and traditional manual counting was performed. In total, 918 FISH images from 320 consecutive invasive breast cancers were analysed and automatically classified into 5 groups according to the 2018 ASCO/CAP guidelines. The overall classification accuracy was 85.33% (157/184) with a mean average precision of 0.735. In Group 5, the most common group, the consistency was as high as 95.90% (117/122), while the consistency was low in the other groups due to the limited number of cases. The causes of this inconsistency, including clustered HER2 signals, coarse CEP17 signals and some section quality problems, were analysed. The developed AI model is a reliable tool for evaluating HER2 amplification statuses, especially for breast cancer in Group 5; additional cases from multiple centres could further improve the accuracy achieved for other groups.

## Introduction

The human epidermal growth factor receptor 2 (HER2) oncogene is located on chromosome 17q12^1^ and encodes a transmembrane tyrosine kinase receptor. Amplification of the HER2 gene occurs in 15–20% of breast cancer patients^2^ and is related to poor disease outcomes^3^. The introduction of the monoclonal antibody trastuzumab has greatly improved the prognoses of patients with HER2-positive breast cancer^4^. In addition, combined with the oestrogen receptor (ER), the progesterone receptor (PR), Ki-67, CK5/6, and the epidermal growth factor receptor (EGFR), HER2 plays an important role in the molecular classification of breast cancer ^5^. Different molecular subtypes might predict different outcomes^6^and may be associated with preferred treatment regimens^5^. Therefore, an accurate HER2 status assessment method is essential for breast cancer patients.

In clinical practice, immunohistochemistry (IHC) is the most widely used method. According to the 2018 American Society of Clinical Oncology (ASCO)/College of American Pathologists (CAP) guidelines, the immunostaining results of HER2 are reported as follows: 0, negative; 1+, negative; 2+, equivocal; and 3+, positive^7^. If an IHC result is 2+, a further HER2 assessment must be carried out by fluorescence in situ hybridization (FISH) with the same specimen or by a new test with another new specimen^7^. For FISH, HER2 amplification statuses are divided into five groups based on the HER2/CEP17 ratio and the average HER2 copy number^7^. Although IHC is efficient and economical, FISH is more objective and quantitative. In addition, FISH is superior in terms of predicting the clinical outcomes of breast cancer^8^. More importantly, HER2 gene amplification can also be found in HER2 IHC 0 or 1+ cases by FISH testing^9,10^. FISH is thus indispensable for HER2 assessment. However, it is undeniable that manually counting HER2 signals is time-consuming and that interobserver variability does exist. A series of automated image analysis methods for HER2 FISH interpretation have emerged over the past two decades, including automated image analysers^11,12^, automated scanning-based imaging approaches (Metasystems^13^ and the Ariol system^14^), the Micrometastasis Detection System (MDS)^15^, and automated signal enumeration systems (CW4000 CytoFISH software^16^ and Vysis AutoVysion^17^). These automated techniques are greatly helpful for HER2 FISH assessments and can provide results that are consistent with manual counting^18^. However, poorly segmented and incorrectly selected cells need to be manually removed^11^, and the automated scoring results produced by HER2 FISH on the Ariol system did not show excellent concordance with pathologists^14^. In addition, automated image analysis requires a large amount of storage power for recording FISH images, potentially resulting in clinical use limitations.

In recent years, deep learning and artificial intelligence (AI) have been greatly developed and utilized in a wide range of fields^19^. Based on a deep learning approach, a previous study established an algorithm for automatically predicting the molecular subtypes and detecting the intratumoral heterogeneity of breast cancer^20^. Additionally, an AI model can predict breast cancer grades, histologic subtypes, ER statuses^21^ and HER2 IHC scores^22^ from haematoxylin and eosin (HE)-stained images. However, studies assessing the HER2 gene amplification statuses of FISH images based on deep learning and AI are scarce^23^.

In the present study, a deep learning-based AI method was established for automated HER2 FISH evaluation. To evaluate the algorithm, breast cancers belonging to five HER2 FISH groups in a testing set were diagnosed by the Aitrox AI model and the gold standard, and their diagnosis results were compared.

## Materials and methods

### Sample selection and HER2 FISH image acquisition

Three hundred and twenty invasive breast cancers determined with both HER2 IHC and FISH detection were collected consecutively from the archives of the Department of Pathology, Fudan University Shanghai Cancer Center (FUSCC). All 320 cases included primary breast cancers (293 cases) and metastatic breast cancer tissues in lymph nodes (17 cases), the lung (3 cases), the liver (3 cases), the chest wall (3 cases), and bone (1 case). All samples were included in the study with approval from the independent ethical committee/institutional review board, and all participants signed informed consent forms. All methods were performed in accordance with the relevant guidelines and regulations. All study activities were conducted in accordance with the Declaration of Helsinki and relevant guidelines and regulations.

Representative 4-μm-thick formalin-fixed paraffin-embedded (FFPE) tumour sections were assessed by two pathologists (TX and QMB) to determine the carcinoma areas using HE-stained sections. Afterwards, the PathVysion HER2 DNA Probe Kit (Abbott Molecular, Abbott Park, Illinois) was used for HER2 FISH according to the manufacturer’s instructions. The specific experimental method for FISH was as follows. The sections were baked at 65 °C for 30 min and dewaxed 3 times in xylene at room temperature for 10 min each time, followed by immersion in 100% ethanol for 5 min. The sections were sequentially placed in 100% ethanol for 2 min, 85% ethanol for 2 min and 70% ethanol for 2 min, and the sections were immersed in pure water at room temperature for 3 min. The sections were treated with pure water at 88–92 °C for 30 min. The pepsin solution was prepared as follows: pepsin powder (activity 1:3000, Solarbio) (75 mg) was dissolved in 150 ml 0.01 HCL with pH ≈ 2.0. After cooling the sections to below 60 °C, they were immersed in pepsin solution and incubated for 15–20 min at 54 °C. The sections were then rinsed once in pure water. The sections were dehydrated in 70% ethanol for 1 min at room temperature. The slides were then dried. The probe working solution was added dropwise to the target hybridization areas of the sections, a coverslip was immediately applied, and the periphery of the coverslip was sealed with rubber cement. The slides were placed in a hybridization apparatus, and a hybridization procedure was set. Denaturation was performed at 80 °C for 8 min, and this was followed by hybridization at 39 °C for 16–18 h. Then, the slide sealant was carefully removed in a dark environment, and the slides were immersed in 0.4× saline sodium citrate solution for approximately 10 min. The slides were placed in a 0.3% NP-40/0.4× saline sodium citrate solution (preheated to 68–72 °C, pH ≈ 7.0), shaken for 1–3 s, and rinsed for 2 min. The slides were incubated at room temperature in 0.1% NP-40/2× saline sodium citrate (pH ≈ 7.0), shaken for 1–3 s, and rinsed for 1 min. The slides were then placed in 70% ethanol at room temperature and rinsed for 1 min. The slides were removed and dried. Approximately 20 µL of DAPI was added dropwise to each slide, followed by a coverslip.

The FISH results obtained for HER2 were independently evaluated by two pathologists (TX and QMB) according to the criteria described in the 2018 ASCO/CAP guidelines^7^. The HER2 FISH results were designated into five groups. The FISH images of tumours were manually selected for scanning. High-quality FISH images were captured using the Microscope Image Information System (MIIS) from Shanghai Aitrox Technology Corporation Limited (Shanghai, China) and saved in JPEG file format.

### Aitrox AI model

Based on a deep convolutional neural network (DCNN), our method, named the Aitrox AI model, consists of two steps for automatically detecting HER2 amplification statuses in FISH images with a tumour cell nucleus detector and a signal detector. In the first step, tumour cell nuclei are localized in a whole FISH image by the tumour cell nucleus detector. The tumour cell nucleus detector has an architecture based on You Only Look Once version 3 (YOLOv3)^24^. YOLOv3 is a state-of-the-art DCNN that is widely used in object detection cases. YOLOv3 consists of two modules: a feature extractor and a predictor. The feature extractor uses a network named Darknet-53 with successive 3 × 3 and 1 × 1 convolutional layers and shortcut connections, and it performs on par with state-of-the-art classifiers and while converging more quickly^24^. The predictor predicts bounding boxes using dimension clusters as anchor boxes at three different scales, which integrates rich feature maps from low to high levels and the classes that the bounding boxes may contain^25^. A whole FISH image goes through the tumour cell nucleus detector, during which bounding boxes with tumour cell nucleus probabilities are predicted. The bounding boxes with higher probabilities (above a specified threshold) are taken as detected tumour cell nuclei.

**Figure 1:**
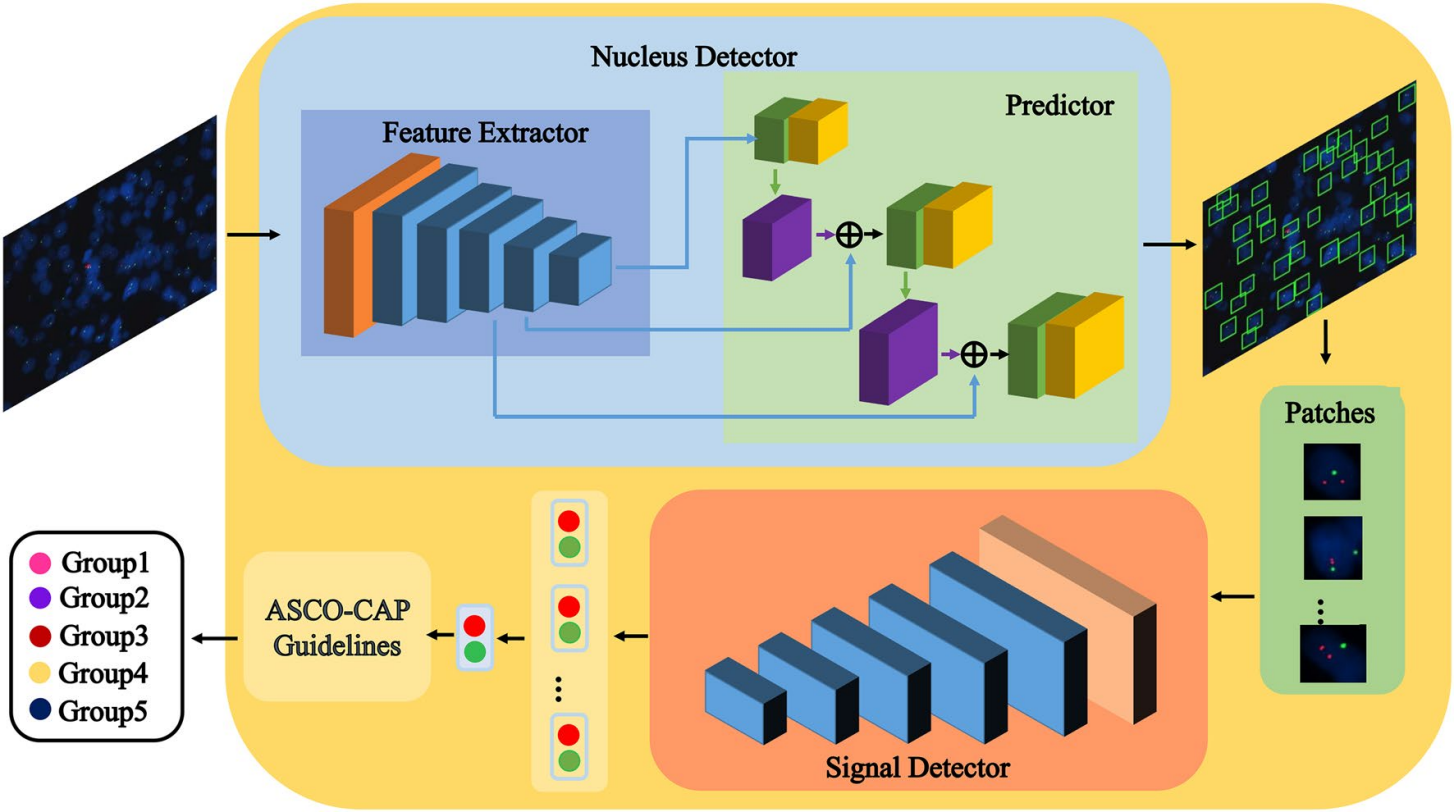
Pipeline of the Aitrox AI model for the automatic detection of HER2 amplification statuses in FISH images. First, a whole FISH image goes through the tumour cell nucleus detector with a feature extractor and predictor to localize the tumour cell nuclei. The detected tumour cell nuclei are cropped from the original image as patches. Second, the signal detector takes patches as input and outputs their HER2 and CEP17 gene signal quantities. HER2 amplification statuses are classified into 5 groups according to the ASCO/CAP guidelines.

In the second step, the numbers of HER2 and CEP17 signals are counted by the signal detector in one detected tumour cell nucleus derived from the tumour cell nucleus detector. ResNet 18^26^, a DCNN with good feature extraction performance, is adopted as the signal detector. The signal detector takes one detected tumour cell nucleus as input and outputs its HER2 and CEP17 gene signal quantities, which is an obvious regression task. Subsequently, to convert the localization and counting results acquired from the tumour cell nucleus detector and the signal detector into image-level predictions, the numbers of HER2 gene signals and CEP17 gene signals in one FISH image are obtained, from which the average HER2 gene copy number is obtained and the HER2/CEP17 ratio is calculated. The HER2 gene copy number equals the ratio of the number of tumour cell nuclei to the number of HER2 gene signals in one FISH image. The HER2/CEP17 ratio is defined as the ratio of the number of CEP17 gene signals to the number of HER2 gene signals within a FISH image. The whole workflow of the proposed deep learning framework is shown in Fig. 1.

### Preprocessing and training procedure

The FISH images were divided into three groups: training, validation and test groups. To train the tumour cell nucleus detector and the signal detector, the FISH images were manually annotated by two pathologists (TX and MR) with two steps corresponding to the two DCNNs. First, bounding box annotations were provided to train the tumour cell nucleus detector. The bounding boxes were comprehensively annotated according to their tumour cell characteristics, such as their nucleus sizes and tissue structures. These bounding boxes had four coordinates for their positions and ranges in images and one single label named the tumour cell nucleus for their class. Second, the numbers of HER2 gene signals and CEP17 gene signals for each bounding box were annotated not only to train the signal detector but also to calculate the HER2 gene copy number and HER2/CEP17 ratio. Within one FISH image, the number of tumour cell nuclei equalled the number of bounding boxes, which could be easily obtained after detecting the tumour cell nuclei with the tumour cell nucleus detector.

During the training procedure, a loss function and optimization were employed to improve the performance of the DCNNs. To better train the tumour cell nucleus detector, the loss function combined the generalized intersection over union (GIoU) loss, objectness loss and classification loss. Since the IoU loss only considers the ratio of the areas of intersection and merging between the real box and the prediction box, when the prediction box and the target box do not intersect, the gradient disappears, and the loss cannot accurately reflect the degree of overlap between the two boxes. We used the GIoU loss to add a penalty term to the IoU loss to alleviate the above problems and make the prediction results more accurate. We used adaptive moment estimation (Adam) optimization for training the tumour cell nucleus detector with a fixed learning rate of 1e−3. Adam combines the advantages of Adagrad, which is good at dealing with sparse gradients, and root-mean-square propagation (RMSprop), which is good at handling nonstationary targets. The parameters of each iteration were adjusted within a certain range so that these parameters remained relatively stable. For the signal detector, we took the mean square error loss as the loss function and Adam as the optimization function with a fixed learning rate of 1e−3. In the pretraining phase, pretrained weights were loaded during the training procedures of both DCNNs. The pretrained weights of the tumour cell nucleus detector were obtained through pretraining on the COCO dataset^27^, while the pretrained weights of the signal detector were obtained via pretraining on the ImageNet dataset^28^. We used rotation, translation, etc., to enhance each input FISH image. Moreover, both DCNNs were trained on one NVIDIA GeForce 1080Ti. The validation set was used to select the best-performing model during the training procedure.

### Ethics approval

This study was approved by the Institutional Review Board of Fudan University Shanghai Cancer Center.

### Consent to participate

Informed consent was obtained from all individual participants included in the study.

## Results

**Figure 2:**
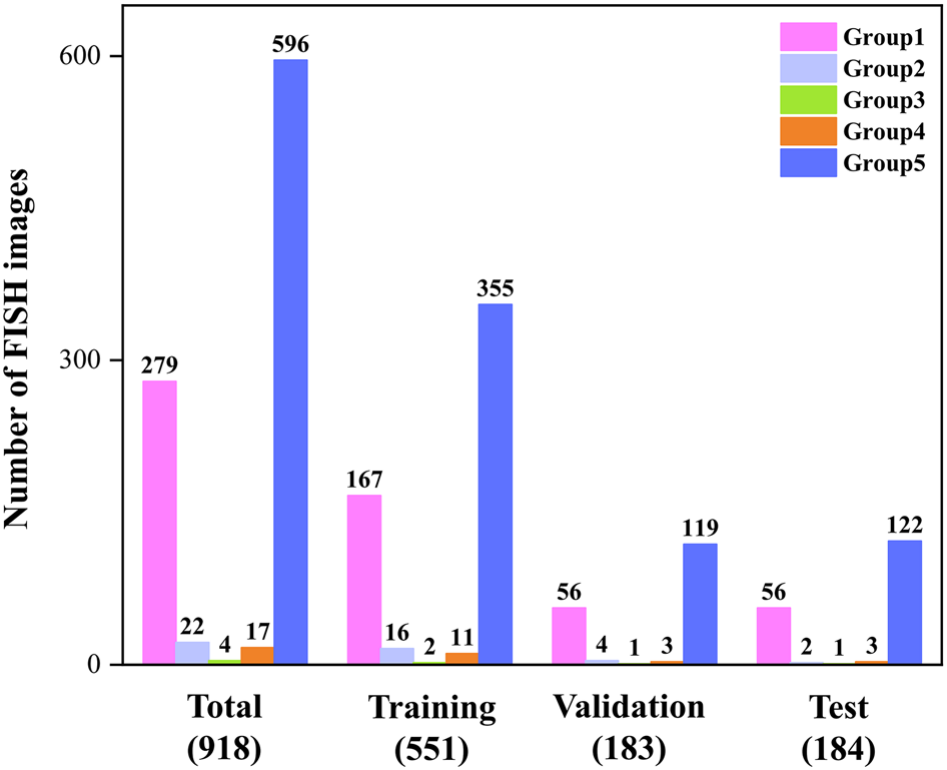
Detailed information on the sample distributions of different datasets.

A total of 918 FISH images scanned from 320 patients were obtained from FUSCC. The FISH images were divided into three sets: training (551/918, 60.02%), validation (183/918, 19.94%) and test sets (184/918, 20.04%). Each patient's FISH images only appeared within a single dataset. For the automated analysis, 4969 and 1275 bounding boxes with tumour cell nucleus labels were obtained in the training set and validation set, respectively. Each set was classified into five groups based on the ASCO/CAP guidelines. Detailed information on the distributions of the 918 FISH images contained in the three sets is shown in Fig. 2. No significant differences were observed among the three sets with respect to clinicopathologic characteristics, especially between the training and test sets (Table 1).

**Table 1:** Clinicopathologic characteristics in training set, validation set and test set.

	Training set	Validation set	Test set	*p* value (training set vs. validation set)	p value (training set vs. test set)	*p* value (validation set vs. test set)
Age (years)						
≤ 50	93	31	36	0.657	0.530	0.900
> 50	87	33	40			
Tumor size (cm)						
< 2	27	8	10	0.284	0.446	0.764
≥ 2	38	19	20			
Tumor grade						
1/2	63	20	27	0.304	0.898	0.434
3	74	33	33			
Node status						
Negative	68	32	25	0.302	0.708	0.603
Positive	41	13	13			
Molecular subtypes						
Luminal A	39	19	15	0.052	0.225	0.403
Luminal B	90	21	29			
HER2-overexpression	21	6	12			
Triple-negative breast cancer	30	18	19			

**Figure 3:**
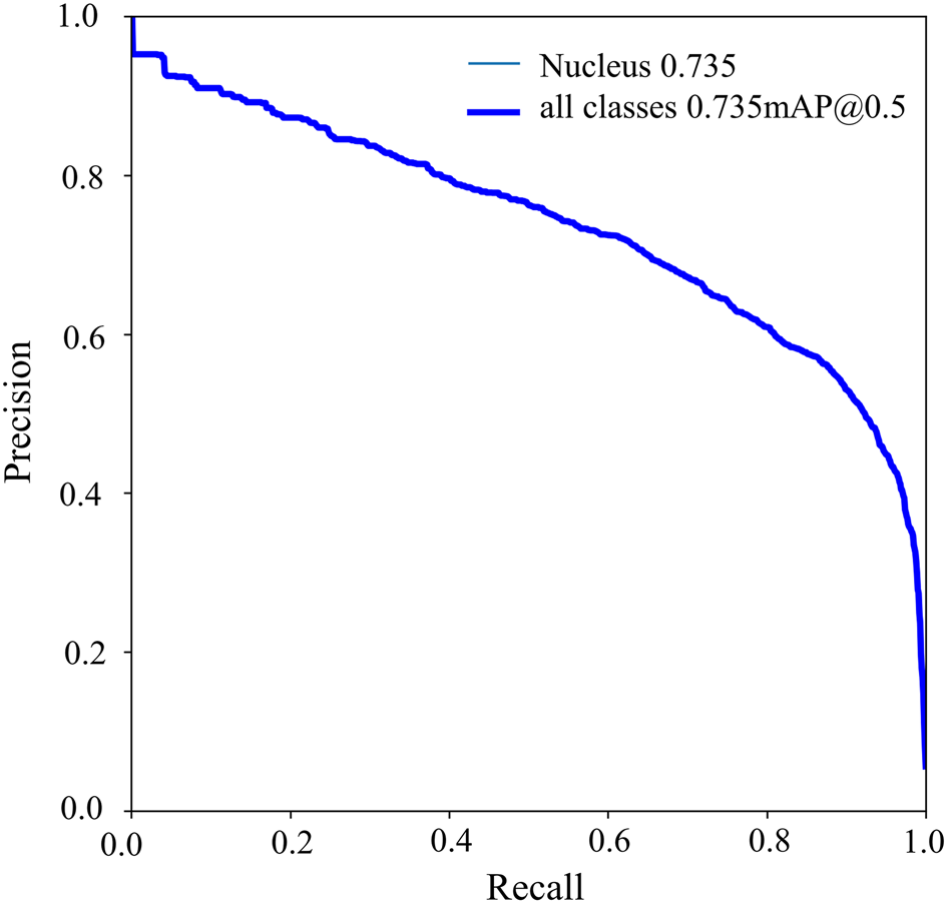
Mean average precision (mAP) curve produced for the test dataset.

**Figure 4:**
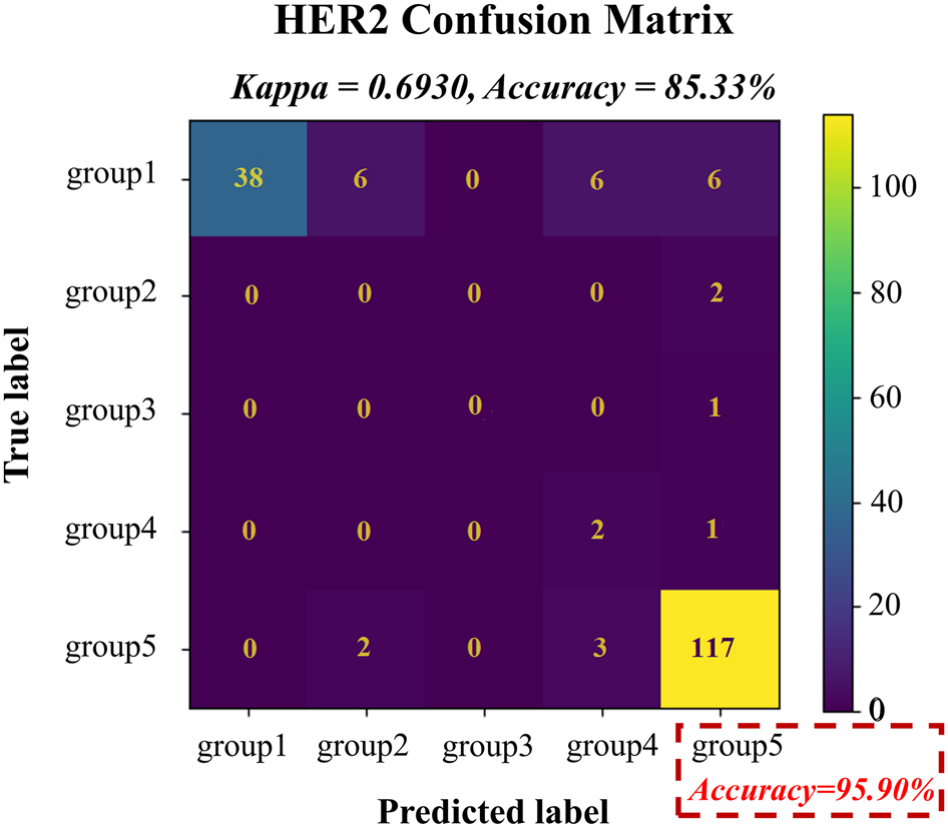
Confusion matrix obtained for the test set concerning the classifications provided by the pathologists and the Aitrox AI model.

The case-based classifier’s performance was tested on 184 FISH images, and it achieved 85.33% (157/184) accuracy. The mean average precision (mAP) curve of the test dataset was obtained through the tumour cell nucleus detector network, and the mAP for the test dataset was 0.735 (Fig. 3). A comparison between the classification results obtained by the pathologists and the Aitrox AI model on the test set is shown in Fig. 4. In Group 1, 67.86% (38/56) of the samples were correctly classified through the Aitrox AI model, while the remaining 32.14% (18/56) were equally classified into Group 2, Group 4 and Group 5. In Group 2, both samples (2/2) were misclassified into Group 5. Group 3 only contained one sample, which was misclassified into Group 5. In Group 4, 66.67% (2/3) of the samples were correctly classified. In Group 5, 95.90% (117/122) of the samples were correctly classified, while the remaining 1.64% (2/122) and 2.46% (3/122) were classified into Group 2 and Group 4, respectively.

**Figure 5:**
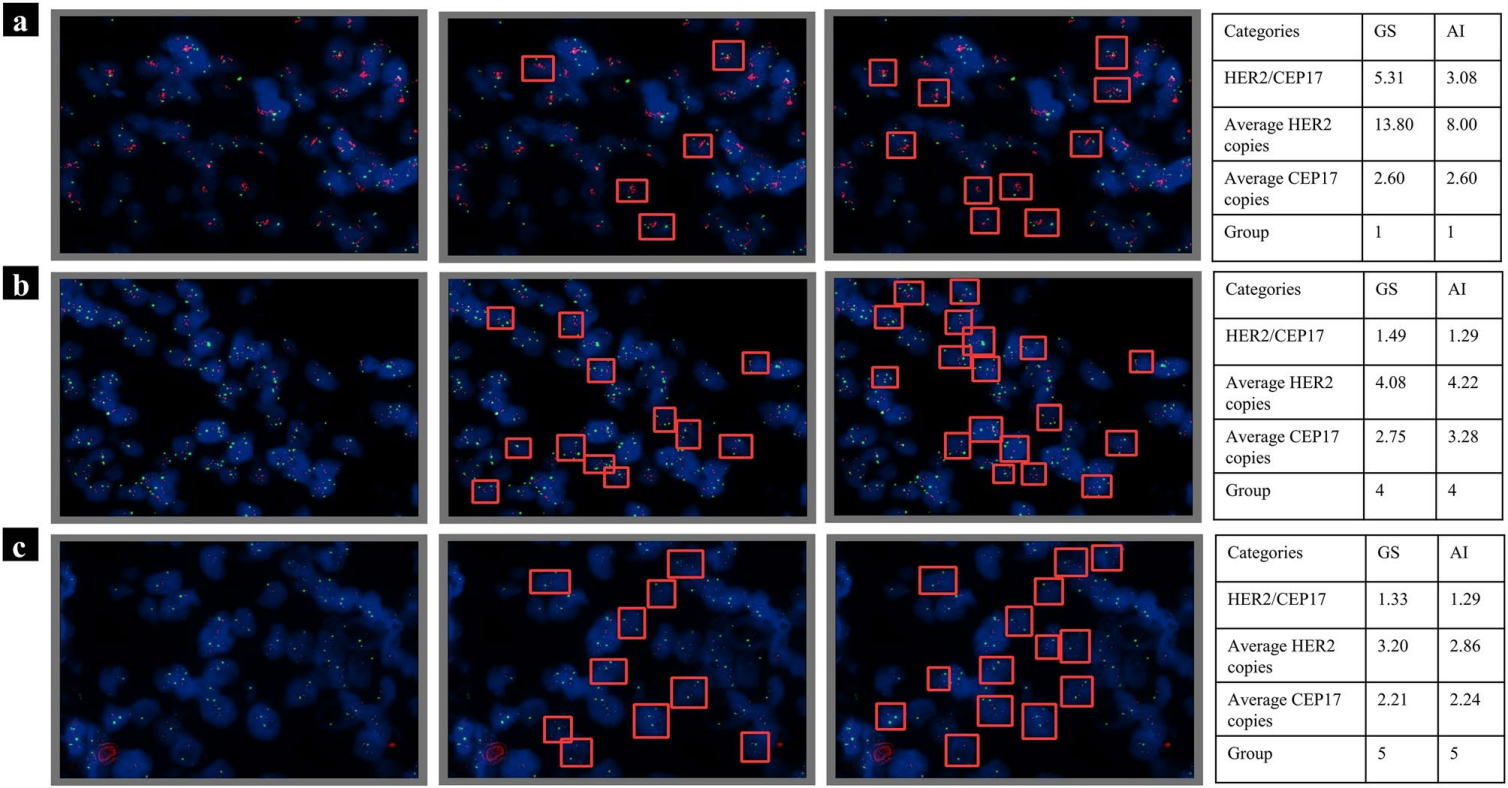
Selected and representative samples from the five groups that were correctly predicted by the Aitrox AI model. (**a**) Group 1. (**b**) Group 4. (**c**) Group 5. The first column shows the original images of the five groups. The second column shows the original images labelled by pathologists, which were used as the gold standard in the test dataset. The third column shows the original images predicted by our Aitrox AI model. The HER2/CEP17 ratio was calculated for both the gold standard and our Aitrox AI model as the ratio of the number of CEP17 gene signals to the number of HER2 gene signals in one image, and details can be found in the last column.

Representative samples from the five groups that were correctly predicted by the Aitrox AI model are shown in Fig. 5. Figure 5a shows the original image, the original image with labelled bounding boxes, and the original image with the AI-predicted bounding boxes for Group 1. The pathologist-labelled and AI-predicted average HER2 copies, CEP17 copies and HER2/CEP17 ratios were 13.80 and 8.00, 2.60 and 2.60, and 5.31 and 3.08, respectively. Figure 5b shows the original image, the original image with labelled bounding boxes, and the original image with the AI-predicted bounding boxes for Group 4. The pathologist-labelled and AI-predicted average HER2 copies, CEP17 copies and HER2/CEP17 ratios were 4.08 and 4.22, 2.75 and 3.28, and 1.49 and 1.29, respectively. Figure 5c shows the original image, the original image with labelled bounding boxes, and the original image with the AI-predicted bounding boxes for Group 5. The pathologist-labelled and AI-predicted average HER2 copies, CEP17 copies and HER2/CEP17 ratios were 3.20 and 2.86, 2.21 and 2.24, and 1.33 and 1.29, respectively.

**Figure 6:**
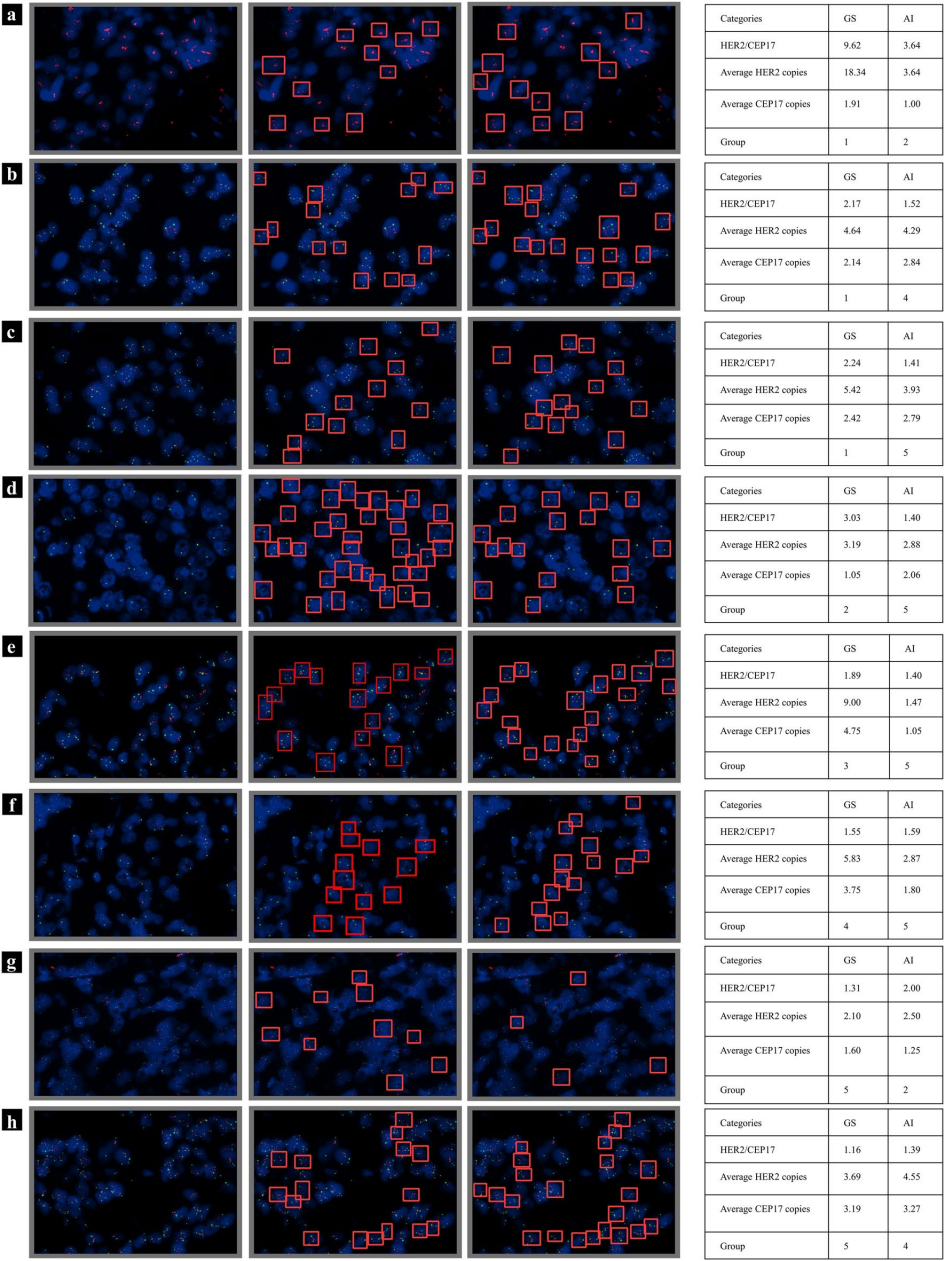
Selected and representative samples from the five groups that were incorrectly predicted by the Aitrox AI model. (**a**–**c**) Group 1. (**d**) Group 2. (**e**) Group 3. (**f**) Group 4. (**g**,**h**) Group 5. The first column shows the original images of the five groups. The second column shows the original images labelled by pathologists, which were used as the gold standard in the test dataset. The third column shows the original images predicted by the Aitrox AI model. The HER2/CEP17 ratio was calculated for both the gold standard and our Aitrox AI model as the ratio of the number of CEP17 gene signals to the number of HER2 gene signals in one image, and details can be found in the last column.

Figure 6 shows selected and representative samples from the five groups that were incorrectly predicted by the Aitrox AI model. Figure 6a–c show the original image, the original image with labelled bounding boxes, and the original image with the AI-predicted bounding boxes for Group 1. However, the AI model incorrectly classified this sample into Group 2, Group 4 and Group 5. Figure 6d–f show the original image, the original image with labelled bounding boxes, and the original image with the AI-predicted bounding boxes for Groups 2, 3 and 4, respectively. However, the AI model incorrectly classified these samples into Group 5. Figure 6g,h show the original image, the original image with labelled bounding boxes, and the original image with the AI-predicted bounding boxes for Group 5. However, the AI model incorrectly classified these samples into Group 2 and Group 4. Detailed information is provided in Fig. 6.

## Discussion

HER2 protein overexpression and gene amplification statuses are crucial markers for evaluating prognoses and making treatment decisions for breast cancer patients. It is promising that the HER2 IHC score could be predicted using automated image analysis^29^, and the experimental results exhibited good concordance with the FISH results^30^. For HER2 FISH interpretation, a computer-aided image analysis method has been developed. The overall accuracy of automated spot counting and manual scoring ranged from 82 to 100%^15–17,31,32^. In addition, studies have demonstrated that image analysis can be performed a significantly shorter evaluation time^17,33^. However, other studies have suggested that image analysis underestimates the HER2 and CEP17 data of FISH images compared with the results of the conventional method^34^, and manual intervention is always required during the image analysis process^17^. In this study, an automated detection method (the Aitrox AI model) for FISH images was constructed through a two-step deep learning pipeline. First, the nuclei of tumours in FISH images were localized and classified by the tumour cell nucleus detector network. Then, the numbers of HER2 and CEP17 gene signals were calculated by the signal detector network, and a final HER2/CEP17 ratio was obtained. This model correctly classified the majority of samples into different HER2 amplification status subgroups, especially for Group 5.

Among the incorrectly classified Group 1 cases, HER2 signals formed clusters in 12 cases, and AI obviously underestimated the HER2 count. In accordance with the presented results, previous studies have demonstrated that clusters are not suitable for regression-based methods; they instead recommended density-based methods^35^. However, undefined results are obtained through density-based methods when no CEP17 signals are available because signal quantification is completed on a single fluorescence channel^35^. In fact, it is easy to identify cases with clustered HER2 signals and to manually draw conclusions in clinical practice. In addition, the numbers of CEP17 signals determined by the AI model were higher than the manual counts in eight misclassified Group 1 cases and in two misclassified Group 2 cases, presumably because the CEP17 signals were slightly coarse. One case each in Group 3 and Group 4 was mistakenly classified into Group 5. In the misclassified images, the HER2 signals in some cells were gathered, and the intensity levels of HER2 signals in some cells were inconsistent, which may have been the reason for the low HER2 signal count produced by the AI method. Among the incorrectly classified Group 5 cases, two cases were mistakenly classified into Group 2. In one case, the weak CEP17 signals may not have been detected by the signal detector, and the reason for the other case was that a few cells were selected by the tumour cell nucleus detector. In the other three cases mistakenly assigned to Group 4, a few clustered nuclei were counted as one nucleus by AI in the two cases, leading to an increased number of HER2 signals. The remaining case may have been caused by counting bias. In histological samples, nuclei often overlap, so their signals cannot be counted exactly within one tumour cell nucleus. A new method was suggested, which counted all dots in representative tumour areas and assessed HER2/CEP17 ratios without considering the nucleus boundaries^18^. However, the HER2 copy number is also important, and HER2/CEP17 ratios cannot be considered alone when evaluating HER2 gene statuses. Therefore, AI may not be suitable for cases containing overlapping nuclei.

The present study has several limitations. In the tumour cell nucleus detector, the tile sampling method is used; however, the size of the tile does not always correspond to the size of a tumour cell nucleus, particularly when nuclei overlap. Nevertheless, tile-sampling analysis still performed well in previous research^17^ as well as in our study. A tumour cell nucleus sampling classifier should be considered in our future work. Furthermore, only a few FISH images were contained in Group 2, Group 3, and Group 4. Our data might be representative of real-world data since our cases were collected consecutively from FUSCC. Nevertheless, we included 918 FISH images in our study, making it the largest dataset in this field to our knowledge. To achieve improved AI classification accuracy for these three groups, further studies should include more cases from multiple centres.

Heterogeneity was also not assessed in our study. Heterogeneity can manifest in multiple patterns. HER2-amplified cells and nonamplified cells can be completely separated or intermingled^36^. Several studies have focused on automatically evaluating HER2 heterogeneity. Nguyen et al.^37^ developed a high-content quantitative analysis method based on microfluidic experimentation and image processing to detect HER2 intratumoral heterogeneity at the cellular level. Radziuviene et al.^34^ reported that an automated high-capacity nonselective tumour cell assay could generate evidence-based HER2 intratumor heterogeneity. However, the complicated experimental procedure in the abovementioned method may limit its clinical application.

Based on these limitations, we propose a method for combining pathologists with the Aitrox AI model, since scanning an entire FISH slice is time-consuming and requires significant storage space, and manual counting is insufficient for accurately counting tumour cells. Pathologists review the FISH sections under a fluorescence microscope (400x) to determine whether the HER2 signals are heterogeneous. If no heterogeneity is observed, several representative tumour regions are manually selected and then submitted to the Aitrox AI model for interpretation. If heterogeneity exists, several representative tumour regions in different groups will be manually selected and then submitted to AI for interpretation. In addition, pathologists could determine whether the HER2 signals form clusters, the signal intensities, and whether the nuclei overlapped during scanning whole-slide images to decide whether the slide is suitable to submit for Aitrox AI model interpretation. More importantly, HER2 amplification statuses could be preliminarily determined during this process. The artificial selection of regions can ensure the inclusion of tumour components. Pathologists can evaluate the AI interpretation result and manually modify it if the result does not meet expectations. Combining the advantages of manual quality control and AI counting, this method can not only ensure the accuracy of pathological reports but also improve the efficiency of pathologists, demonstrating great clinical practicability.

In conclusion, the Aitrox AI model is a reliable tool for automatically evaluating HER2 amplification statuses, especially for breast cancers in Group 5. Further studies involving more cases in other groups from multiple centres are still needed.

## Data Availability

The training, validation and test datasets utilized for the development of the deep-learning model cannot be publicly available due to the need to protect the privacy and health information of the patients involved. However, the datasets can be provided upon request if the Institutional Review Board of the ethics committee of Fudan University Shanghai Cancer Center (FUSCC) grants approval. The corresponding author (QMB) should be contacted if someone wants to request the data from this study.
